# An early economic evaluation of active surveillance for low-risk ductal carcinoma *in situ*

**DOI:** 10.1080/14796694.2024.2421152

**Published:** 2024-12-16

**Authors:** Danalyn Byng, Michael Schaapveld, Esther H Lips, Frederieke H van Duijnhoven, Jelle Wesseling, Wim H van Harten, Valesca P Retèl

**Affiliations:** a Division of Psychosocial Research & Epidemiology, The Netherlands Cancer Institute-Antoni van Leeuwenhoek Hospital, Amsterdam, CX 1066, The Netherlands; b Erasmus School of Health Policy & Management, Erasmus University Rotterdam, Rotterdam, PA 3062, The Netherlands; c Division of Molecular Pathology, The Netherlands Cancer Institute-Antoni van Leeuwenhoek Hospital, Amsterdam, CX 1066, The Netherlands; d Division of Surgical Oncology, The Netherlands Cancer Institute-Antoni van Leeuwenhoek Hospital, Amsterdam, CX 1066, The Netherlands; e Department of Pathology, Leiden University Medical Center, Leiden, ZA 2333, The Netherlands

**Keywords:** active surveillance, biomarkers, cost–effectiveness, ductal carcinoma in situ, early economic evaluation

## Abstract

**Aim:** Perform early economic evaluation comparing active surveillance (AS) to surgery for women with low-risk ductal carcinoma *in situ*, a precursor of invasive breast cancer.

**Materials & methods:** A 10-year incremental costs (€) and quality-adjusted life years (QALYs) were compared between a simulated cohort of women undergoing breast conserving surgery ± radiotherapy, and a cohort with a low-risk subgroup undergoing AS using a semi-Markov model. Scenario and headroom analyses evaluated a better-performing biomarker to select low-risk women for AS.

**Results:** AS resulted in lower costs and survival, but higher QALYs (±0.40). Scenario analyses maintained survival outcomes and maximized QALYs.

**Conclusion:** AS for low-risk ductal carcinoma *in situ* is cost-effective, but a better-performing biomarker to select low-risk women can maximize quality-adjusted outcomes.

## Abstract (plain language summary)

Biomarkers to identify women who are at low-risk of a subsequent invasive breast cancer after ductal carcinoma *in situ* (DCIS) are still in the exploration phase.

There is potential for a biomarker-driven active surveillance (AS) strategy to be an alternative approach to surgery for low-risk DCIS.

We characterize the costs and quality-adjusted health outcomes associated with (non)-interventional strategies using a semi-Markov model for early cost–effectiveness modeling.

We explore two bio-marker based strategies for selecting low-risk women who could opt for an AS strategy. First, we use standard pathological information on DCIS grade (low-to-intermediate) and estrogen-receptor-positive status, similar to the eligibility criteria of ongoing prospective clinical trials for AS. In the scenario analysis, models used information on COX-2 protein expression and breast adipocyte size to select low-risk women to forgo surgery.

Scenario and headroom analyses were performed to evaluate a hypothetical better-performing biomarker using information on COX-2 protein expression and breast adipocyte size to select the low-risk subgroup.

Forgoing surgery among these women resulted in significant gains in quality of life, despite an expected elevated rate of ipsilateral invasive breast cancer and somewhat reduced life years on average.

This early economic evaluation demonstrated that introducing an AS option to select women with low-risk features can be a cost-effective alternative to immediate surgery and adjuvant radiotherapy.

A better-performing biomarker to select low-risk women can maximize quality-adjusted outcomes.

## 1. Background & objectives

Active surveillance (AS) is a disease management strategy that allows for the routine monitoring of a given condition for signs of progression. The aim is to uphold quality of life and avoid interventional treatment and related side effects unless warranted following a progression of the condition. A now widely accepted strategy for some men with low-risk prostate cancer [1], AS is currently being evaluated as an alternative to surgical resection in (ongoing) clinical trials for women diagnosed with low-risk primary ductal carcinoma *in situ* (DCIS), a potential precursor of invasive breast cancer (IBC), in the LORD (LOw Risk DCIS), LORIS (LOw Risk dcIS) and COMET (comparing an operation to monitoring, with or without endocrine therapy) studies [2–5].

The motivation to bring AS into clinical practice for DCIS stems from an understanding about the heterogeneous risk of subsequent ipsilateral IBC (iIBC) following a diagnosis of primary DCIS [6]. For women with low-risk DCIS features, including low-to-intermediate grade and estrogen receptor [ER]-positive status, their risk of progression to iIBC remains low [7] enough to make them an ideal group to consider the option of AS. As these women make up approximately 50% of screen-detected DCIS [8], the impact on the healthcare system could be considerable.

With a high prevalence of DCIS (representing 20–25% of all screen-detected ‘breast cancers’) the present costs of treatment to the healthcare system are substantial [9]. The current standard of care dictated by professional guidelines in Europe and the USA recommend surgical resection of the lesion, possibly followed by radiotherapy and endocrine therapy for all DCIS [10,11]. Treatment-related morbidity and the related impact on health-related quality of life among the many women now living with a diagnosis of primary DCIS has already motivated the movement toward de-escalation strategies for adjuvant therapy [12]. Multigene assays such as the Oncotype DX DCIS score and DCISionRT are used to select women who could forgo radiotherapy after breast conserving surgery (BCS) [13,14]. While these have been clinically validated, neither option has been found to be cost-effective [15–17]. For “good-risk” patients defined by the Radiation Therapy Oncology Group (RTOG) 9804 study (≥60 years, ER-positive, tumor extent 2.5 cm, low-to-intermediate grade, and margins ≥3 mm), adjuvant radiotherapy and tamoxifen use were associated with reduced ipsilateral breast recurrence over the long-term follow-up period [18]. However, despite the risk-reducing effect of both radiotherapy and tamoxifen, observation after BCS was found to be the most cost-effective option for women with these “good-risk” features [19,20].

Recent DCIS cohort studies have identified further promising prognostic factors which have strong associations with developing subsequent iIBC: human epidermal growth factor receptor (HER2) overexpression (odds ratio (OR) 1.56; 95% confidence interval (95% CI), 1.05–2.31), high cyclooxygenase (COX)-2 protein expression (OR: 2.97; 95% CI: 1.72–5.10), presence of periductal fibrosis (OR: 1.44; 95% CI: 1.01–2.06), and large breast adipocyte size (OR: 2.75; 95% CI: 1.25–6.05) [21,22]. DCIS with both high COX-2 expression and large breast adipocytes was associated with a 12-fold higher risk (OR: 12.0; 95% CI: 3.10–46.3) for subsequent iIBC [22].

With an increasing understanding of the heterogeneous nature of DCIS, the possibility to further select women who can safely forgo locoregional treatment based on these features or markers is promising. While these prognostic factors await specification and validation in larger clinical studies, BCS will remain the minimum accepted approach for all women with DCIS.

In the Netherlands, women with newly diagnosed DCIS with low-risk features participating in a discrete choice experiment demonstrated strong preferences to forgo BCS and adjuvant therapy altogether, opting instead for AS despite the current minimal clinical evidence to support the safety and feasibility of such a strategy [23]. Results from prospective AS trials will however not be available for at least 10 years. Using mathematical modelling techniques, real-world cancer registry data, and DCIS patient-derived quality of life and preference information, the objective of this paper is to simulate possible patient- and health-system level impacts of introducing an AS strategy. In addition, we can simulate scenarios using different possible biomarkers to improve the selection of women eligible for AS. This early economic model can inform future research and policy and foreshadow the likely drivers of cost–effectiveness associated with presumed trial outcomes. Furthermore, the maximum possible cost for a hypothetical perfect biomarker solution can be modeled.

## 2. Materials & methods

### 2.1. Comparators

**Figure 1.**
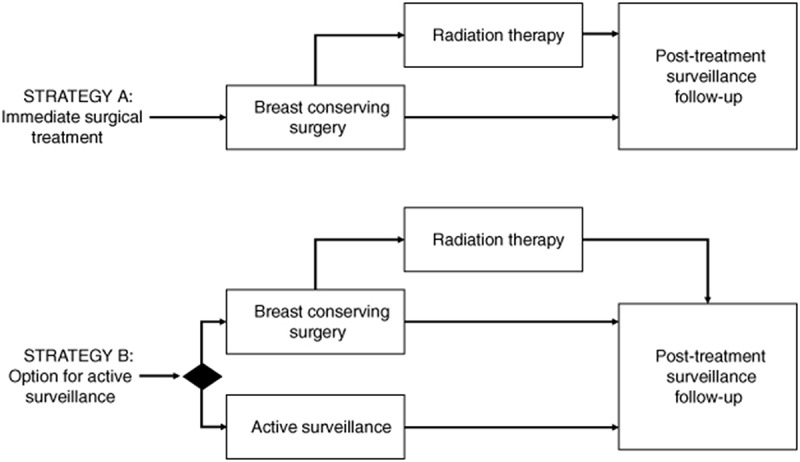
Strategies explored in the economic evaluation. The base-case model compares two strategies for all women with screen-detected primary DCIS. Strategy A consists of standard immediate surgical treatment for all DCIS, consisting of breast conserving surgery with (75%) or without (25%) radiotherapy, followed by annual surveillance mammography for 5 years post diagnosis. The comparator strategy (Strategy B) similarly focuses on all women with screen-detected DCIS, while using a biomarker (indicated by the diamond decision node) to identify a subset of women at low-risk for progression to ipsilateral invasive breast cancer. Fifty percent of the cohort would be eligible for active surveillance based on low-risk characteristics (estrogen receptor positive DCIS grade I/II). Seventy-five percent of women treated with BCS will undergo adjuvant radiation therapy. AS: Active surveillance; BCS: Breast conserving surgery; DCIS: Ductal carcinoma *in situ*.

The base-case model compares two strategies for all women with screen-detected primary DCIS (Figure 1). The first strategy (Strategy A) consists of standard immediate surgical treatment for all DCIS following European clinical guidelines, consisting of BCS with (75%) or without (25%) radiotherapy, followed by annual surveillance mammography for 5 years post-diagnosis [8]. The comparator strategy (Strategy B) similarly focuses on all women with screen-detected DCIS, while using standard pathological information to identify a subset of women at low-risk for progression to ipsilateral IBC. Standard pathological information on the DCIS grade (low-to-intermediate) and ER-positive status, similar to the eligibility criteria in the LORD trial, is used to identify low-risk women. These women are considered eligible to forgo surgery and opt for an AS strategy consisting of clinical follow-up and surveillance mammography for 10 years post-diagnosis. Based on observed patterns of enrolment into the LORD trial and previously reported Dutch registry data, we assumed 50% of all women with screen-detected primary DCIS have low-risk features and would be eligible for the AS strategy [8].

### 2.2. Setting & location

The decision model is set in the Netherlands and takes on a Dutch healthcare perspective considering only direct medical costs. A 10-year time-horizon was chosen to limit the dependency on assumptions, given lack of availability of head-to-head trial or real-world data on the comparative effectiveness of surgery versus AS. This time horizon was further substantiated by the evidence that the use of adjuvant RT is only associated with lower risk of iIBC in the first decade after DCIS diagnosis, with lower risks of second breast events over time [24,25]. The persistence of the treatment effect of surgery on survival beyond 10 years is also not known [26]. It is expected that in 10-years time, technological advancements will have been made which can more accurately select women for AS.

### 2.3. Choice of model

A multistate modeling approach was used to simulate the disease process after diagnosis with primary DCIS. This approach is based on a continuous-time semi-Markov model [27,28]. The use of Markov models to conduct economic evaluations in healthcare settings is widespread, but may be more appropriate to model disease processes for chronic, long-term illnesses given the use of constant unvarying transition probabilities and the memoryless “clock-forward” property [29]. For our purposes modeling DCIS, we use a semi-Markov model which employs a clock-reset approach [30]. History, or time spent in a given state is measured, allowing transition probabilities between states to be time-dependent. Survival times from which transition probabilites are derived are treated as continuous variables. Such a model also allows for the consideration of population heterogeneity and competing event and mortality risks [31].

**Figure 2.**
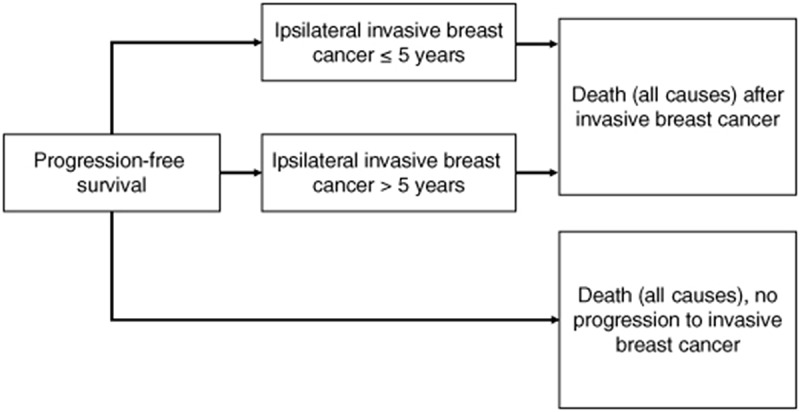
Multi-state model. A graphic representation of the semi-Markov model, also known as a multistate model, used to simulate the disease process after a primary DCIS diagnosis. Women begin at the progression-free survival state, and can transition to iIBC within, or after 5 years, or eventually transition to death without experiencing an iIBC. DCIS: Ductal carcinoma *in situ*; iIBC: Ipsilateral invasive breast cancer.

The semi-Markov multistate model was built with five health states: iIBC-free; iIBC within 5 years of DCIS diagnosis; iIBC more than 5 years post-DCIS diagnosis; death after iIBC; and death without experiencing iIBC (Figure 2). The iIBC health state was split into two to capture the different possible biological processes relating to a subsequent iIBC. This decision was due to the observed difference in frequency of events (hazard rate) in the first 5 years, compared with after 5 years [25,32]. Two all-cause death states are modeled, occurring either after an intermediate iIBC event, or without any intermediate event.

### 2.4. Target population & subgroups

The source population of this study was a retrospective cohort of screening-age women diagnosed with primary DCIS derived from the surveillance, epidemiology, and end results program (SEER) cancer registry database. The purpose of using such a population-based cancer registry for information on treatment uptake, DCIS clinicopathological characteristics, and outcomes was to ensure a representative cohort of women who would be considered for a AS strategy, with a representative distribution of characteristics. Furthermore, a large subgroup of untreated women was only feasible to derive from SEER, and not from Dutch registry sources. Using the SEER cancer registry database, we identified N = 31,068 women aged 45–75 at the time of DCIS diagnosis. Women with low-risk characteristics (low-to-intermediate grade, ER+) were identified as the low-risk subgroup.

In order to ensure that subsequent iIBCs were abstracted correctly as new primaries, we selected women who were diagnosed with DCIS from 2007 onwards, given the changes in SEER coding rules that required registrars to record subsequent invasive breast cancer following DCIS as a new primary cancer and not a locoregional invasive recurrence [33]. In the selected cohort, women were diagnosed up to and including 2016. The cohort included women who were treated for their DCIS with breast conserving surgery, with or without radiotherapy, as well as women who did not undergo immediate treatment with surgery.

This cohort is a subset of a larger previously-reported DCIS cohort; covariate selection and missing data imputation steps are described elsewhere [32]. DCIS treatment is modeled based on patterns of care for surgery and radiotherapy observed in SEER for this cohort.

### 2.5. Treatment strategy outcomes

In this decision model, treatment outcomes for women undergoing surgery are based on patient-level outcomes observed directly in the SEER cohort. Conditional transition probabilities were computed by building Cox proportional hazards models stratified by transition to compute cumulative transition hazards transformed into conditional transition probabilities using the Aalen–Johansen estimator [34]. State occupation probabilities at different time points following DCIS diagnosis could be derived from these values. State occupation and transition probabilities are derived separately for women with low-risk (low-to-intermediate grade, ER+) and normal/high risk DCIS characteristics who underwent surgery. To model outcomes for the subset of women opting for the AS strategy, patient-level iIBC outcomes observed in SEER for the subgroup of untreated women with low-risk characteristics were modeled. The remaining transition probabilities from the iIBC states and for all-cause death were taken from the surgery group. Data preparation and multi-state modeling was done using the R package mstate version 0.2.11. Probabilities are provided in the Supplementary Appendix: probability of remaining in DCIS state (Supplementary Table S1); state transition probabilities from DCIS state to iIBC (Supplementary Table S2); state transition probabilities from DCIS state to death (Supplementary Table S3); state transition probabilities from iIBC ≤5 years post-DCIS diagnosis to death (Supplementary Table S4); state transition probabilities from iIBC >5 years post diagnosis to death (Supplementary Table S5).

### 2.6. Health-related quality of life

Health-related quality of life and utility measurements were derived from two studies evaluating health-state and treatment preferences among women with DCIS. Utilities were derived from a study which quantified preferences for managing (low-risk) screen detected DCIS using the EQ-5D-5L approach among women with a personal history of DCIS [35]. This is the only study published to-date which has derived utility values directly from women with DCIS. A utility decrement for salvage mastectomy was derived from a cost–effectiveness study on adjuvant treatment for low-risk DCIS reported by Ward et al. [19]. It is assumed that the utility weights associated with progression remain the entire occupancy time in a given state, up to the full time horizon of 10 years [35].

### 2.7. Estimating resources & costs

In this model, we assumed that all women surgically-treated for their DCIS will undergo breast conserving surgery and 75% of these women will undergo adjuvant radiotherapy. Women who experience an invasive breast cancer event after their DCIS diagnosis, who were previously treated with breast conserving surgery with or without radiotherapy, are treated with salvage mastectomy, (immediate) breast reconstruction with implant, and adjuvant chemotherapy in line with to European and American clinical guidelines on locoregional recurrence following DCIS [11,36–38]. Women following the AS strategy who experience a subsequent invasive breast cancer are primarily treated with breast conserving surgery, adjuvant radiotherapy and chemotherapy.

Associated healthcare utilization and surgical costs were based on Dutch costs derived from a previously published population-based cost-utility analysis of four common surgical treatment pathways for breast cancer in the Netherlands [39]. This analysis followed a large, representative cohort of women, and included the cost of the surgical intervention, outpatient visits, admission days, diagnostics-related resources and costs of complications during the treatment. Chemotherapeutic costs for women who experience an invasive breast cancer event are based on a previously published Dutch cost–effectiveness model for women with early-stage breast cancer [40]. All costs were reported in 2020 Euros, with any adjustments made using the Consumer Price Index.

### 2.8. Deterministic analysis

Total costs and quality-adjusted life years (QALYs) were discounted at 4.0 and 1.5%, respectively, according to the Dutch Guidelines for the Conduct of Economic Evaluations in HealthCare [41]. The incremental cost–effectiveness ratio (ICER) was calculated as the difference in costs divided by the difference in QALYs between the cohort including an AS strategy, and the cohort following standard interventional treatment.

### 2.9. Sensitivity analyses

One-way sensitivity analyses were performed to assess robustness of model outcomes. Cost parameters were individually assessed at the minimum and maximum values of their range (Table 1) to identify those most influential on incremental costs. Utility parameters were similarly assessed based on the accompanying upper and lower bounds of the reported 95% confidence interval to identify influence over incremental QALYs.

**Table 1.** Base-case model parameters.

Cost	Base cost (EUR)	Range (EUR)	Distribution	Ref.
Breast conserving surgery, including re-excision	9,636	7,227–12,045	Gamma	[39]
Whole breast radiotherapy	7,606	5,704–9,508	Gamma	[39]
Salvage mastectomy	9,553	7,165–11,941	Gamma	[39]
Immediate implant-based reconstruction following mastectomy	19,554	14,666–24,442	Gamma	[39]
Chemotherapeutic treatment^a^	16,600	12,450–20,750	Gamma	[40]
Mammography	91.97	68.98–114.96	Gamma	[42]

Utility	Base Estimate	Range	Distribution	Ref.
Breast conserving surgery alone	0.768	0.696–0.848	Beta	[35]
Breast conserving surgery with radiotherapy	0.729	0.592–0.837	Beta	[35]
Active surveillance	0.879	0.848–1.000	Beta	[35]
Progressed DCIS to invasive breast cancer	0.622	0.388–0.745	Beta	[35]
Utility decrement of receiving salvage mastectomy	0.180	0.080–0.250	Beta	[19]

All oncologic surgery costs include non-OR, outpatient, admission, diagnostics and plastic surgery costs.

a Includes chemotherapy costs, infection-prevention medication, outpatient stay costs.

### 2.10. Probabilistic analyses

Probabilistic analyses were used to simultaneously assess the uncertainty of all inputs by randomly drawing cost and utility parameter values from assigned distributions. Beta distributions were used for utilities, and gamma distributions were used for costs. Five thousand Monte Carlo simulation iterations were used. The results of the simulations are illustrated in an incremental cost–effectiveness plane, which visualizes the extent to which including an option for AS is more or less effective and expensive compared with standard immediate interventional treatment. The effectiveness of AS is shown separately as incremental life years and as QALYs gained/lost among all women with DCIS.

Cost–effectiveness acceptability curves were plotted to illustrate the impact of uncertainty on the outcomes, given a range of possible willingness to pay (WTP) thresholds per QALY gained. In the Netherlands, a threshold of €80,000 per QALY is standard for severe diseases, for preventive strategies a lower WTP level of €20.000 is used [43]. Across the Monte Carlo simulation iterations, we report incremental discounted costs, life years, QALYs and accompanying 95% credential intervals (CI) for each strategy.

### 2.11. Scenario analysis

Scenario analysis models used information on COX-2 protein expression and breast adipocyte size to select low-risk women to forgo surgery, based on the case-control study of Almekinders et al. [22]. According to the study, women with low COX-2 protein expression and with lower relative area of breast adipose tissue (adipocyte area^75th^) had a cumulative incidence of iIBC similar to the general population. While the study population received BCS only, following expert consultation, the scenario analysis assumes no added value of surgery, and that iIBC incidence remains similar to the general population.

In this scenario analysis, these women (representing ~10% of the screen-detected DCIS population) would be eligible to forgo surgery. Under an AS strategy their risk of iIBC is assumed to remain the same (i.e., BCS is assumed to have no effect), and are therefore considered low-risk. Normal-risk women would be treated with BCS only. Using data on a a subset of screening-age women from the study [22], we derived transition probabilities for the DCIS to iIBC states for the low-risk subgroup (those with low COX-2 protein expression and adipocyte area^75th^) and for the remaining normal-risk DCIS population. All other transition probabilities to the remaining health states were derived from the base-case model, using only data on women treated with BCS. A comparison is made to a standard-care strategy, where all women are treated with BCS regardless of risk, and 75% receive adjuvant radiotherapy.

### 2.12. Comparison of low-risk patients by biomarker

In order to better visualize and compare the variability across Monte Carlo simulations, probabilistic results and cost–effectiveness planes are reported separately for the two subsets of women eligible for AS: low-risk women based on strategy B in the base-case model (low-to-intermediate grade, ER+) and low-risk women based on the scenario analysis (low COX-2 protein expression and adipocyte area^75th^). Incremental QALYs per patient in each biomarker scenario are reported.

### 2.13. Headroom analysis for hypothetical perfect biomarker to select low-risk women

A headroom analysis [44] was conducted to estimate the maximum cost for which a (new) biomarker can be brought to market while maintaining the cost–effectiveness of its use. This analysis focuses on the biomarkers defined in the scenario analysis: COX-2 protein expression and breast adipocyte size. They are considered ‘hypothetically perfect’ because outcomes in the low-risk group are assumed to mirror the treated population, as illustrated by Almekinders et al. [22]. As this scenario analysis simulated a biomarker with optimal diagnostic accuracy, we used the results to conduct a headroom analysis to determine the maximum possible cost for a hypothetical perfect biomarker solution that would accurately select women who could forgo surgery (assumed to be 10% of entire cohort). For the cost of the new technology to remain cost-effective, the headroom is assessed as the net reduction of costs associated with the strategy, plus the societal WTP threshold for a QALY multiplied by the maximum QALY gain across the entire cohort [44,45].

$$\begin{array}{c} \mathrm{Headroom}=\left(\mathrm{Net}\;\mathrm{reduction}\;\mathrm{of}\;\mathrm{healthcare}\;\mathrm{costs}\right)\\ +\left(\mathrm{WTP}\;\mathrm{threshold}\right)\;\times \left(\mathrm{Additional}\;\mathrm{QALYs}\;\mathrm{generated}\right) \end{array}$$

Analyses were performed using Microsoft Excel, version 2019 (Microsoft, Redmond, WA) and R (R Project, Vienna, Austria). This report conforms to the Consolidated Health Economic Evaluation Reports standards statement [46].

## 3. Results

### 3.1. Base-case model

Table 2 presents results from the base-case model with 100,000 simulated DCIS patients for each strategy. When an AS strategy is introduced for all low-risk (low-to-intermediate grade and ER+) patients as an alternative to immediate surgery, a QALY gain of 0.4 is achieved for the entire DCIS cohort, with an accompanying average per patient cost saving of 6,353€ over the time horizon. Without adjustment for utilities, this strategy results on average in a limited loss of life years (-0.06, 95% CI: -0.26–0.16).

**Table 2.** Probabilistic results.

Strategy	Incremental costs, 95% CI	Incremental life years, 95% CI	Incremental QALYs, 95% CI	ICER costs per QALY
**Base case:** Whole DCIS cohort, 10 year time horizon. Simulation based on SEER data. **Strategy A:** Standard surgical intervention (BCS ± RT) for all women **vs** **Strategy B:** Women with ER+, Grade I/II DCIS (50% of whole cohort) undergo AS; remaining undergo standard surgical intervention (BCS ± RT)	-6,353€ (-8,811, -3,966€)	-0.06 (-0.26, 0.16)	0.40 (-0.15, 0.87)	-15,981€
**Scenario analysis:** Whole DCIS cohort, 10-year time horizon. Simulation based on SEER data and Almekinders et al. **Strategy A:** Standard surgical intervention (BCS ± RT only) for all women **vs** **Strategy B:** Women with Low COX2/Adipocyte area^75th^ Quartile 1 DCIS (10% of whole cohort) undergo AS; remaining undergo standard surgical intervention with BCS only	-7,076€ (-11,978, -2,427€)	0.00 (-0.49, 0.48)	0.23 (-0.30, 0.76)	-31,071€

**Figure 3.**
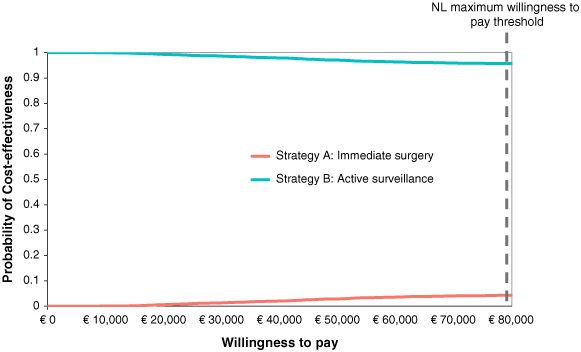
Cost–effectiveness acceptability curve (base–case analysis). Percentage of time each strategy is cost-effective at varying willingness to pay thresholds are represented. The red line represents the percentage of time the standard immediate surgery (Strategy A) is cost-effective, the blue line for the active surveillance strategy (Strategy B). In the Netherlands, a threshold of €80,000 per QALY is standard for severe diseases (grey dashed line), for preventive strategies a lower WTP level of €20.000 is used. QALY: Quality-adjusted life year.

**Figure 4.**
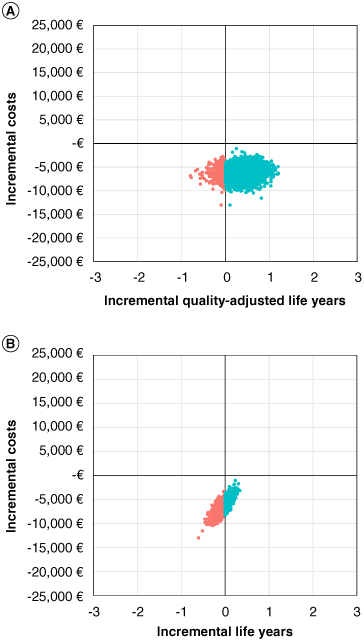
Base-case cost–effectiveness planes for incremental quality-adjusted life years **(A)** and incremental life years **(B)**. Cost–effectiveness planes in the base-case analysis. Each dot on the graph represents the results of the 5000 Monte Carlo simulation iterations for the probabilistic sensitivity analysis of introducing an active surveillance strategy for women with low-risk DCIS (ER+ and low-/intermediate-grade) compared with standard surgical intervention. In **(A)**, effectiveness is represented on the x-axis by incremental QALY, and **(B)** by incremental LY. Points shown in blue represent simulations in which introducing an active surveillance strategy resulted in overall absolute health benefits across the simulated cohort. 93% of simulations showed an increase in incremental QALY **(A)**, 31% of simulations showed an increase in incremental LY **(B)**. AS: Active surveillance; LY: Life year; QALY: Quality-adjusted life year.

The Monte Carlo probabilistic sensitivity analysis demonstrated that introducing an AS strategy produced higher net benefits (i.e., was cost-effective) in 95.7% of the 5000 model simulations at the 80,000€ WTP threshold, and 99.3% at the 20,000€ WTP threshold (Figure 3). This is similarly illustrated in the cost–effectiveness plane in Figure 4A. All model simulations showed a decrease in incremental costs when introducing AS, while 93% of the simulations showed an increase in incremental QALYs. The trend of improved effectiveness was less apparent when considering life years alone: 69% of the simulations showed a decrease in incremental life years (Figure 4B).

Supplementary Figure S1 shows that for all cost inputs, not one drives cost–effectiveness of the AS strategy. Breast conserving surgery, followed by radiotherapy does however have the largest influence over incremental costs. With decreasing costs of each surgical treatment type, the incremental cost savings for the strategy including active surveillance obviously lessens. Conversely, as more women would experience an iIBC in Strategy B, lowering the cost of chemotherapy would also increase the cost savings for this strategy. In Supplementary Figure S2, varying all utilities individually by their lower and upper bounds would still result in a QALY gain for Strategy B. Notably, the utility associated with invasive breast cancer did not have an impact on the cost–effectiveness of introducing an AS strategy.

### 3.2. Scenario analysis

The results of the scenario analysis are visualized in Supplementary Figure S3. A de-escalation strategy whereby 10% of women are selected for AS based on a different definition of low-risk status (low COX-2 protein expression and adipocyte area^75th^) and the remaining undergo BCS, is cost-effective compared with BCS with or without radiotherapy for all women. Compared with the base-case analysis, there is more apparent uncertainty in the model inputs which is indicated by the dispersion of the results of the simulation iterations across the two lower quadrants of Supplementary Figure S3.

### 3.3. Comparison of low-risk patients by biomarkers

Supplementary Figure S4 & Supplementary Table S6 show the results of the Monte Carlo simulation iterations for each of the low-risk subgroups explored in the base-case and scenario analyses. 92.8% of model runs resulted in incremental QALY gains for low-risk women defined by low-to-intermediate grade, ER+ DCIS. The model using low COX-2 expression and adipocyte area^75th^ to define low-risk status had greater parameter uncertainty due to the smaller available dataset [22]. However, 97.0% of model runs resulted in incremental QALY gains for this group of low-risk women. As a group, women following the scenario analysis strategy gained 1.02 QALYs (95% CI: -0.05–1.98), while women in the base-case strategy gained 0.81 QALYs (95% CI: -0.26–1.70).

### 3.4. Headroom analysis

The use of the hypothetical perfect biomarker to select women for the AS strategy was expected to yield an average cost savings of €7076 and 0.23 incremental QALYs per patient based on the scenario analysis for the total DCIS cohort (Table 2).

Headroom=€7,076+€20,000×0.23

Assuming that this biomarker strategy could reliably select 10% of the DCIS cohort as low-risk, the headroom available for this strategy was calculated as €6227 for a WTP threshold of €20,000. This is the maximum unit cost for which this biomarker strategy can be brought to market while remaining cost-effective.

## 4. Discussion

DCIS is a potential precursor to invasive breast cancer. At present, nearly all women with DCIS are treated in the same manner as early breast cancer as it is not possible to reliably predict who may progress to invasive disease. Due to this uncertainty many women with harmless DCIS are treated. This early economic evaluation demonstrated that introducing an AS option to select women with low-risk features can be a cost-effective alternative to immediate surgery and adjuvant radiotherapy. Women with low-to-intermediate grade, ER+ DCIS make up approximately 50% of screen-detected primary DCIS. In this analysis, forgoing surgery resulted in significant gains in quality of life, despite an expected elevated rate of iIBC and somewhat reduced life years in this group.

The results of this and other published studies [19,20,47] on locoregional treatment for DCIS highlight the potential for a range of de-escalation strategies. However, the analyses presented here are limited by the use of data from different sources and different country settings. SEER data was chosen to model outcomes after DCIS given the volume of data available and the rigorous coding rules set forth in 2007 requiring subsequent iIBC to be recorded as new primaries. Despite the availability of data from retrospective DCIS cohorts in the Netherlands [48,49], SEER data has been used extensively to model the natural disease history of DCIS [50,51] and the impact of DCIS treatment, and was therefore chosen for this study. In the future, when patient-level data on AS becomes available, a patient-level simulation can be conducted to better capture the heterogeneity within the population, reflecting the diversity of patient characteristics and treatment responses. Such a level of granularity would enhance a cost–effectiveness model's ability to simulate realistic scenarios, understand variability in outcomes, and provide more accurate insights into the cost–effectiveness of noninterventions for DCIS. Nevertheless, this current study remains an early cost–effectiveness analysis which may have limited generalizability and applicability at this time.

We observed substantial variation in incremental QALYs across simulation iterations. This can be reflective of the variation in preferences regarding benefits to be gained from undergoing AS. The overall positive impact of quality-adjustment does become particularly apparent in the one-way sensitivity analysis (Supplementary Figure S2). Even when applying the lowest value associated with the 2.5th percentile of the utilities’ assigned distributions, resulting incremental QALYs always remained greater than 0.1. These results may foreshadow the forthcoming results of the Dutch LORD study if the 10-year risk of iIBC remains within the prespecified noninferiority threshold.

Whether AS will eventually become an acceptable alternative to surgery still remains uncertain. While half of women with screen-detected primary DCIS would be eligible based on the low-risk criteria of the base-case model, women’s preferences and their access to regular surveillance imaging will dictate whether such a strategy is suitable to them. A discrete choice experiment among Dutch women with newly diagnosed low-risk DCIS found very strong preferences for an active surveillance strategy with no surgery, irrespective of the 10-year risk of iIBC [23]. This preference was also highly related to the desire for consistent follow-up with annual mammography.

A biomarker which selects a more defined group of women who stand to gain no benefit from surgical intervention could alleviate some of the uncertainty and variation around the benefits of an AS strategy. We used a scenario analysis to explore how such a hypothetically perfect biomarker could be used in combination with a de-escalation strategy for all women with screen-detected primary DCIS. The biomarker is considered ‘perfect’ because iIBC rates in the low-risk group would match a healthy population, as demonstrated by Almekinders et al. [22]. The scenario analysis showed a higher QALY gain among this group: 1.02 incremental QALYs compared with 0.81 QALYS among the low-risk group defined in the base case analysis.

In this scenario analysis, a smaller proportion of women (10%) were selected for AS based on COX-2 expression and small adipocyte size, and the remaining women underwent de-escalation to BCS only. The choice of modeling this prevalent de-escalation strategy was intended to address the questions surrounding overtreatment across all women with DCIS. Incremental life years remained unaffected in the scenario analysis, reflecting results from previously published observational cohort studies and randomized controlled trials demonstrating that while RT may reduce rates of local recurrence, it provides no improvement of overall-, distant-metastasis-free or cancer-specific-survival [52–56]. Previously published cost–effectiveness analyses of adjuvant RT for low-risk DCIS similarly demonstrated cost savings for a BCS only strategy; adding RT would result in negligible QALY gains at significant costs [19,20]. While all analyses focused on direct medical costs, there are likely to be significant societal costs associated with treatment of DCIS. Besides direct nonmedical costs associated with caregiver time, there are indirect costs of treatment for DCIS including costs due absenteeism and reduced productivity while working. A study considering societal costs would provide a more holistic perspective capturing the full range of economic consequences associated with DCIS treatment strategies, but is beyond the scope of this study.

One benefit of conducting early cost–effectiveness analyses is that it creates an opportunity to understand the potential of new strategies under the most optimistic assumptions. We performed a headroom analysis based on a simulation of the impact of a hypothetical perfect biomarker using COX-2 and adipose area information. If this biomarker information could indeed accurately select a group of women who could forgo surgery without negatively impacting life years, the potential downstream effects are significant. For such a biomarker-based strategy to remain cost-effective at a WTP threshold of €20,000, the upper ceiling price for this could be set at €6227.

A major shift in contemporary thinking about disease management for DCIS is underway [12,57,58]. Existing literature detailing the heterogeneous nature of DCIS now supports a more individualized approach based on individual risk of progression to invasive breast cancer. But before AS is brought into clinical practice, robust evidence from the LORD, LORIS and COMET trials must first confirm the safety of this approach. This study contributes to the knowledge base on DCIS management, and supports the continuation of research on identifying and validating biomarkers that can select women to safely forgo surgery.

## 5. Conclusion

Active surveillance for a subgroup of women with low-risk DCIS is cost-effective (higher QALYs and lower costs), but may have an even more favorable quality-adjusted outcomes when using a biomarker which performs better in selecting low risk individuals.

## 6. Future perspective

Women with primary DCIS who have a low-risk of experiencing subsequent invasive breast cancer will benefit from an AS strategy instead of active locoregional treatment. Accurate biomarkers for DCIS surgery de-escalation are still in the exploration phase, but early economic evaluations have revealed promise in terms of cost–effectiveness and in the willingness of women to undergo AS.

## Supplementary Material

Supplemental Material
