# Supplementary material for: An early economic evaluation of active surveillance for low-risk ductal carcinoma in situ
Source: Future Oncol. 2024 Dec 16;20(40):3451–62. doi: 10.1080/14796694.2024.2421152 (PMC12219084; doi:10.1080/14796694.2024.2421152)
Supplement: Supplemental Material [file IFON_A_2421152_SM0395.docx]

SUPPLEMENTARY APPENDIX

**An early economic evaluation of active surveillance for low-risk DCIS**

CONTENTS

[Supplementary Table 1. Probability of remaining in DCIS state (deterministic state occupancy of progression free survival). Base case: whole DCIS cohort, 10-year time horizon. Simulation based on SEER data. 3](#_Toc151660766)

[Supplementary Table 2. Deterministic state transition probabilities from DCIS state to ipsilateral invasive breast cancer. Probabilities for transitions in the first 5 years and last 5 years are derived through separate models. Base case: whole DCIS cohort, 10-year time horizon. Simulation based on SEER data. 4](#_Toc151660767)

[Supplementary Table 3. Deterministic state transition probabilities from DCIS state to death (all causes) without experiencing progression to ipsilateral invasive breast cancer. Base case: whole DCIS cohort, 10-year time horizon. Simulation based on SEER data. 5](#_Toc151660768)

[Supplementary Table 4. Deterministic state transition probabilities from ipsilateral invasive breast cancer (≤5 years post-DCIS diagnosis) to death (all causes). Base case: whole DCIS cohort, 10-year time horizon. Simulation based on SEER data. 6](#_Toc151660769)

[Supplementary Table 5. Deterministic state transition probabilities from ipsilateral invasive breast cancer (>5 years post-DCIS diagnosis) to death (all causes). Base case: whole DCIS cohort, 10-year time horizon. Simulation based on SEER data. 7](#_Toc151660770)

[Supplementary Figure 1. One-way sensitivity analysis for costs 8](#_Toc151660771)

[Supplementary Figure 2. One-way sensitivity analysis for utilities 9](#_Toc151660772)

[Supplementary Figure 3. Cost-effectiveness planes (scenario analysis) 10](#_Toc151660773)

[Supplementary Figure 4. Cost-effectiveness planes for low-risk subgroups only 11](#_Toc151660774)

[Supplementary Table 6. Probabilistic results, low-risk subgroups only 12](#_Toc151660775)

# Supplementary Table 1. Probability of remaining in DCIS state (deterministic state occupancy of progression free survival). Base case: whole DCIS cohort, 10-year time horizon. Simulation based on SEER data.

|  | Strategy A: Standard surgical intervention (BCS±RT) for all women | | | | Strategy B: Women with ER+, Grade I/II DCIS (50% of whole cohort) undergo AS; remaining undergo standard surgical intervention (BCS±RT) | | | |
| --- | --- | --- | --- | --- | --- | --- | --- | --- |
|  | Low-risk DCIS patients treated with BCS±RT | | Normal-risk DCIS patients treated with BCS±RT | | Low-risk DCIS patients undergo AS | | Normal-risk DCIS patients treated with BCS±RT | |
| Year 0 | 1.000 | 0.000 | 1.000 | 0.000 | 1.000 | 0.000 | 1.000 | 0.000 |
| Year 1 | 0.996 | 0.001 | 0.995 | 0.001 | 0.973 | 0.008 | 0.995 | 0.001 |
| Year 2 | 0.990 | 0.001 | 0.988 | 0.001 | 0.954 | 0.010 | 0.988 | 0.001 |
| Year 3 | 0.981 | 0.001 | 0.978 | 0.001 | 0.941 | 0.012 | 0.978 | 0.001 |
| Year 4 | 0.972 | 0.001 | 0.967 | 0.002 | 0.930 | 0.014 | 0.967 | 0.002 |
| Year 5 | 0.959 | 0.002 | 0.955 | 0.002 | 0.917 | 0.015 | 0.955 | 0.002 |
| Year 6 | 0.947 | 0.002 | 0.944 | 0.003 | 0.895 | 0.018 | 0.944 | 0.003 |
| Year 7 | 0.933 | 0.003 | 0.931 | 0.003 | 0.874 | 0.022 | 0.931 | 0.003 |
| Year 8 | 0.920 | 0.003 | 0.918 | 0.004 | 0.851 | 0.027 | 0.918 | 0.004 |
| Year 9 | 0.905 | 0.004 | 0.903 | 0.005 | 0.806 | 0.040 | 0.903 | 0.005 |
| Year 10 | 0.892 | 0.006 | 0.871 | 0.020 | 0.806 | 0.040 | 0.871 | 0.020 |

# Supplementary Table 2. Deterministic state transition probabilities from DCIS state to ipsilateral invasive breast cancer. Probabilities for transitions in the first 5 years and last 5 years are derived through separate models. Base case: whole DCIS cohort, 10-year time horizon. Simulation based on SEER data.

|  | Strategy A: Standard surgical intervention (BCS±RT) for all women | | | | Strategy B: Women with ER+, Grade I/II DCIS (50% of whole cohort) undergo AS; remaining undergo standard surgical intervention (BCS±RT) | | | |
| --- | --- | --- | --- | --- | --- | --- | --- | --- |
|  | Low-risk DCIS patients treated with BCS±RT | | Normal-risk DCIS patients treated with BCS±RT | | Low-risk DCIS patients undergo AS | | Normal-risk DCIS patients treated with BCS±RT | |
| Year 0 | 0.000 | 0.000 | 0.000 | 0.000 | 0.000 | 0.000 | 0.000 | 0.000 |
| Year 1 | 0.002 | 0.000 | 0.002 | 0.000 | 0.014 | 0.005 | 0.002 | 0.000 |
| Year 2 | 0.004 | 0.000 | 0.006 | 0.001 | 0.022 | 0.007 | 0.006 | 0.001 |
| Year 3 | 0.006 | 0.001 | 0.010 | 0.001 | 0.025 | 0.008 | 0.010 | 0.001 |
| Year 4 | 0.009 | 0.001 | 0.014 | 0.001 | 0.033 | 0.010 | 0.014 | 0.001 |
| Year 5 | 0.012 | 0.001 | 0.018 | 0.001 | 0.033 | 0.010 | 0.018 | 0.001 |
| Year 6 | 0.014 | 0.001 | 0.020 | 0.001 | 0.038 | 0.005 | 0.020 | 0.001 |
| Year 7 | 0.018 | 0.001 | 0.023 | 0.001 | 0.045 | 0.012 | 0.023 | 0.001 |
| Year 8 | 0.021 | 0.001 | 0.025 | 0.002 | 0.045 | 0.012 | 0.025 | 0.002 |
| Year 9 | 0.024 | 0.002 | 0.029 | 0.003 | 0.045 | 0.012 | 0.029 | 0.003 |
| Year 10 | 0.030 | 0.003 | 0.032 | 0.003 | 0.045 | 0.012 | 0.032 | 0.003 |

# Supplementary Table 3. Deterministic state transition probabilities from DCIS state to death (all causes) without experiencing progression to ipsilateral invasive breast cancer. Base case: whole DCIS cohort, 10-year time horizon. Simulation based on SEER data.

|  | Strategy A: Standard surgical intervention (BCS±RT) for all women | | | | Strategy B: Women with ER+, Grade I/II DCIS (50% of whole cohort) undergo AS; remaining undergo standard surgical intervention (BCS±RT) | | | |
| --- | --- | --- | --- | --- | --- | --- | --- | --- |
|  | Low-risk DCIS patients treated with BCS±RT | | Normal-risk DCIS patients treated with BCS±RT | | Low-risk DCIS patients undergo AS | | Normal-risk DCIS patients treated with BCS±RT | |
| Year 0 | 0.000 | 0.000 | 0.000 | 0.000 | 0.000 | 0.000 | 0.000 | 0.000 |
| Year 1 | 0.003 | 0.000 | 0.003 | 0.001 | 0.013 | 0.005 | 0.003 | 0.001 |
| Year 2 | 0.006 | 0.001 | 0.006 | 0.001 | 0.024 | 0.008 | 0.006 | 0.001 |
| Year 3 | 0.012 | 0.001 | 0.012 | 0.001 | 0.033 | 0.009 | 0.012 | 0.001 |
| Year 4 | 0.019 | 0.001 | 0.020 | 0.001 | 0.037 | 0.010 | 0.020 | 0.001 |
| Year 5 | 0.029 | 0.002 | 0.027 | 0.002 | 0.051 | 0.012 | 0.027 | 0.002 |
| Year 6 | 0.039 | 0.002 | 0.035 | 0.002 | 0.067 | 0.015 | 0.035 | 0.002 |
| Year 7 | 0.049 | 0.002 | 0.045 | 0.003 | 0.081 | 0.018 | 0.045 | 0.003 |
| Year 8 | 0.059 | 0.003 | 0.057 | 0.003 | 0.104 | 0.024 | 0.057 | 0.003 |
| Year 9 | 0.071 | 0.004 | 0.068 | 0.004 | 0.149 | 0.039 | 0.068 | 0.004 |
| Year 10 | 0.079 | 0.005 | 0.098 | 0.020 | 0.149 | 0.039 | 0.098 | 0.020 |

# Supplementary Table 4. Deterministic state transition probabilities from ipsilateral invasive breast cancer (≤5 years post-DCIS diagnosis) to death (all causes). Base case: whole DCIS cohort, 10-year time horizon. Simulation based on SEER data.

|  | Strategy A: Standard surgical intervention (BCS±RT) for all women | | | | Strategy B: Women with ER+, Grade I/II DCIS (50% of whole cohort) undergo AS; remaining undergo standard surgical intervention (BCS±RT) | | | |
| --- | --- | --- | --- | --- | --- | --- | --- | --- |
|  | Low-risk DCIS patients treated with BCS±RT | | Normal-risk DCIS patients treated with BCS±RT | | Low-risk DCIS patients undergo AS | | Normal-risk DCIS patients treated with BCS±RT | |
| Year 0 | 0.000 | 0.000 | 0.000 | 0.000 | 0.000 | 0.000 | 0.000 | 0.000 |
| Year 1 | 0.000 | 0.000 | 0.000 | 0.000 | 0.000 | 0.000 | 0.000 | 0.000 |
| Year 2 | 0.039 | 0.039 | 0.000 | 0.000 | 0.039 | 0.039 | 0.000 | 0.000 |
| Year 3 | 0.039 | 0.039 | 0.017 | 0.017 | 0.039 | 0.039 | 0.017 | 0.017 |
| Year 4 | 0.041 | 0.041 | 0.051 | 0.025 | 0.041 | 0.041 | 0.051 | 0.025 |
| Year 5 | 0.041 | 0.041 | 0.070 | 0.028 | 0.041 | 0.041 | 0.070 | 0.028 |
| Year 6 | 0.044 | 0.044 | 0.090 | 0.031 | 0.044 | 0.044 | 0.090 | 0.031 |
| Year 7 | 0.047 | 0.047 | 0.105 | 0.034 | 0.047 | 0.047 | 0.105 | 0.034 |
| Year 8 | 0.047 | 0.047 | 0.126 | 0.039 | 0.047 | 0.047 | 0.126 | 0.039 |
| Year 9 | 0.047 | 0.047 | 0.180 | 0.063 | 0.047 | 0.047 | 0.180 | 0.063 |
| Year 10 | 0.047 | 0.047 | 0.180 | 0.063 | 0.047 | 0.047 | 0.180 | 0.063 |

# Supplementary Table 5. Deterministic state transition probabilities from ipsilateral invasive breast cancer (>5 years post-DCIS diagnosis) to death (all causes). Base case: whole DCIS cohort, 10-year time horizon. Simulation based on SEER data.

|  | Strategy A: Standard surgical intervention (BCS±RT) for all women | | | | Strategy B: Women with ER+, Grade I/II DCIS (50% of whole cohort) undergo AS; remaining undergo standard surgical intervention (BCS±RT) | | | |
| --- | --- | --- | --- | --- | --- | --- | --- | --- |
|  | Low-risk DCIS patients treated with BCS±RT | | Normal-risk DCIS patients treated with BCS±RT | | Low-risk DCIS patients undergo AS | | Normal-risk DCIS patients treated with BCS±RT | |
| Year 0 | 0.000 | 0.000 | 0.000 | 0.000 | 0.000 | 0.000 | 0.000 | 0.000 |
| Year 1 | 0.000 | 0.000 | 0.000 | 0.000 | 0.000 | 0.000 | 0.000 | 0.000 |
| Year 2 | 0.000 | 0.000 | 0.000 | 0.000 | 0.000 | 0.000 | 0.000 | 0.000 |
| Year 3 | 0.000 | 0.000 | 0.000 | 0.000 | 0.000 | 0.000 | 0.000 | 0.000 |
| Year 4 | 0.000 | 0.000 | 0.000 | 0.000 | 0.000 | 0.000 | 0.000 | 0.000 |
| Year 5 | 0.000 | 0.000 | 0.000 | 0.000 | 0.000 | 0.000 | 0.000 | 0.000 |
| Year 6 | 0.077 | 0.071 | 0.000 | 0.000 | 0.077 | 0.071 | 0.000 | 0.000 |
| Year 7 | 0.104 | 0.074 | 0.048 | 0.045 | 0.104 | 0.074 | 0.048 | 0.045 |
| Year 8 | 0.104 | 0.074 | 0.089 | 0.059 | 0.104 | 0.074 | 0.089 | 0.059 |
| Year 9 | 0.140 | 0.079 | 0.089 | 0.059 | 0.140 | 0.079 | 0.089 | 0.059 |
| Year 10 | 0.181 | 0.085 | 0.089 | 0.059 | 0.181 | 0.085 | 0.089 | 0.059 |

# Supplementary Figure 1. One-way sensitivity analysis for costs


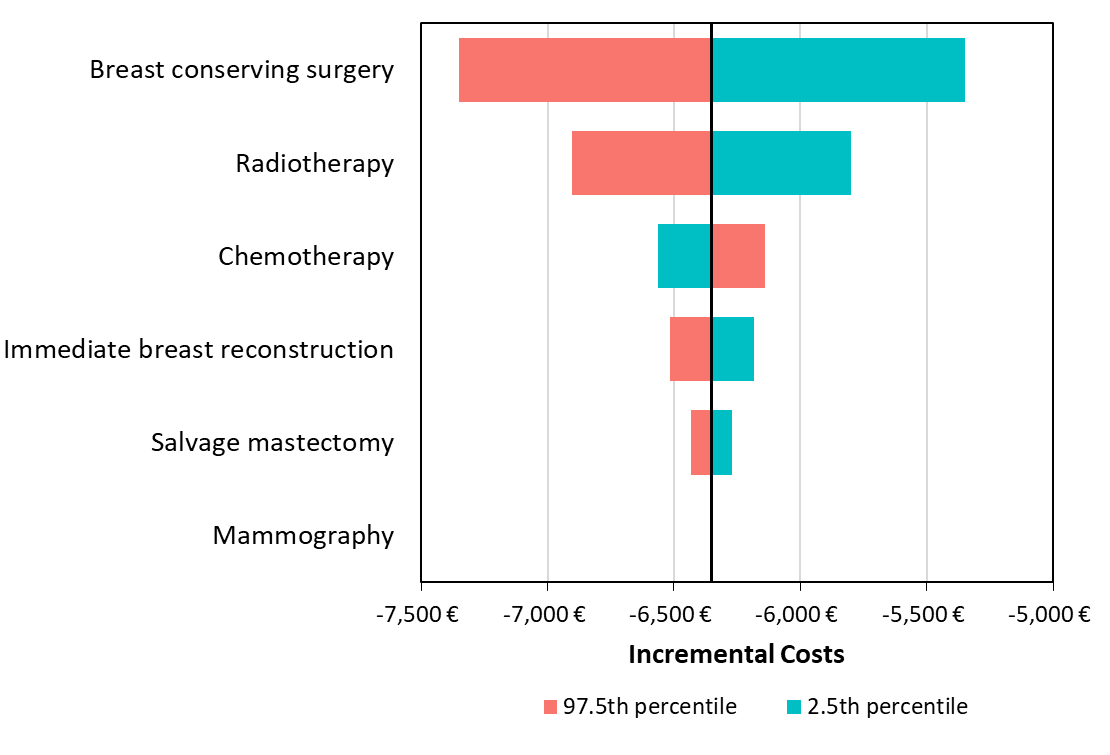


Tornado diagram showing one-way sensitivity analysis of base-case incremental costs to variations in parameters. Parameters are varied one at a time by the 2.5 and 97.5th percentile of their assigned distribution. Ranking is based on size of effect on incremental costs of introducing an active surveillance strategy.

# Supplementary Figure 2. One-way sensitivity analysis for utilities


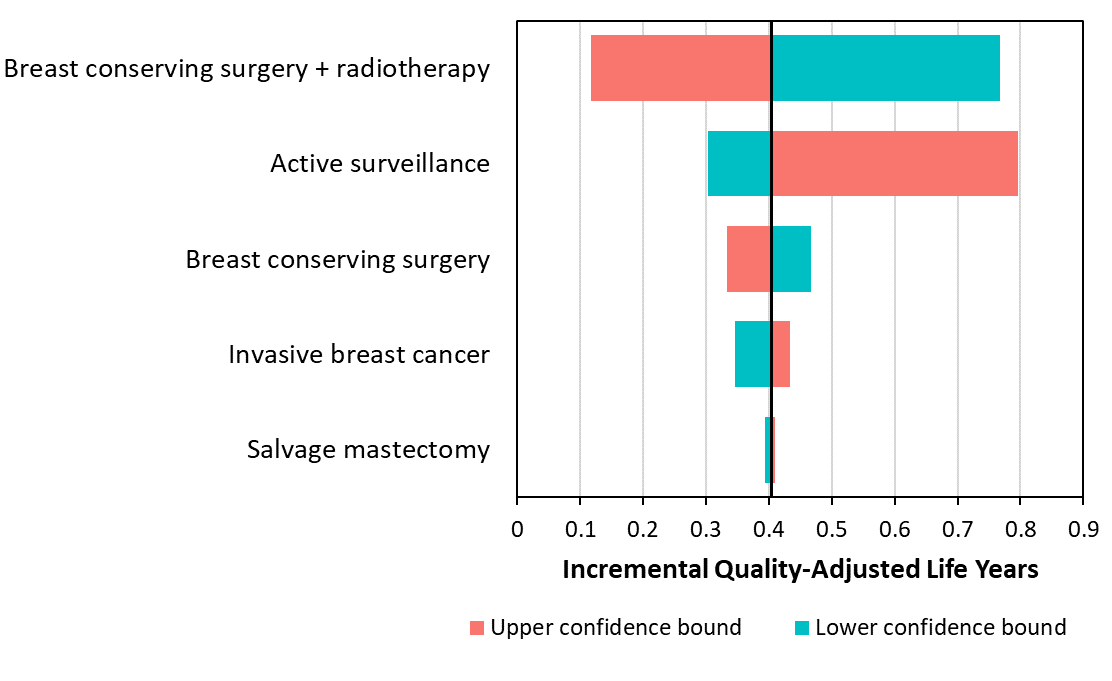


Tornado diagram showing one-way sensitivity analysis of base-case incremental quality-adjusted life years (QALYs) to variations in input parameters. Parameters are varied one at a time by the 2.5 and 97.5th percentile of their assigned distribution. Inputs are ranked by size of effect on incremental QALYs of introducing an active surveillance strategy.

# Supplementary Figure 3. Cost-effectiveness planes (scenario analysis)


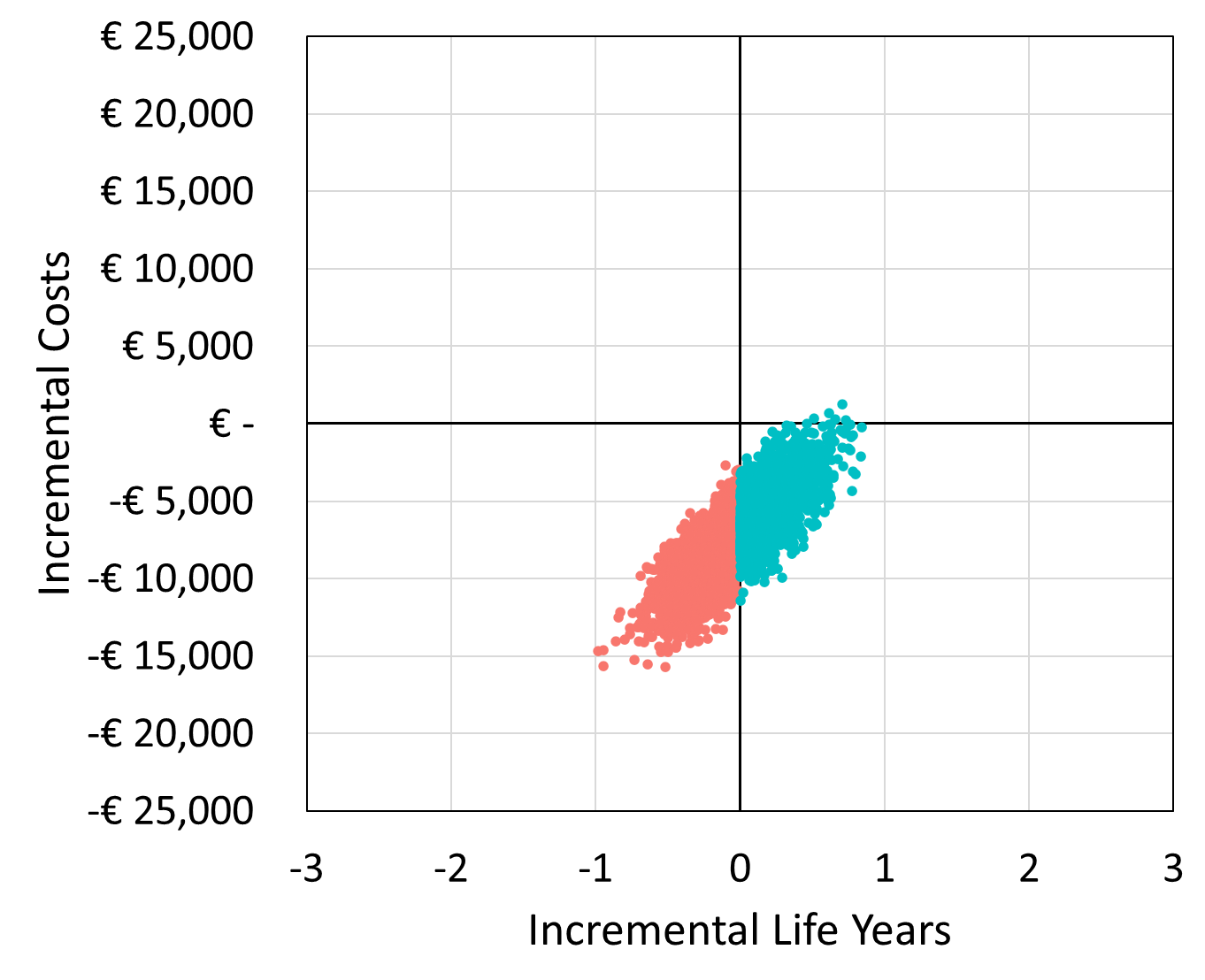

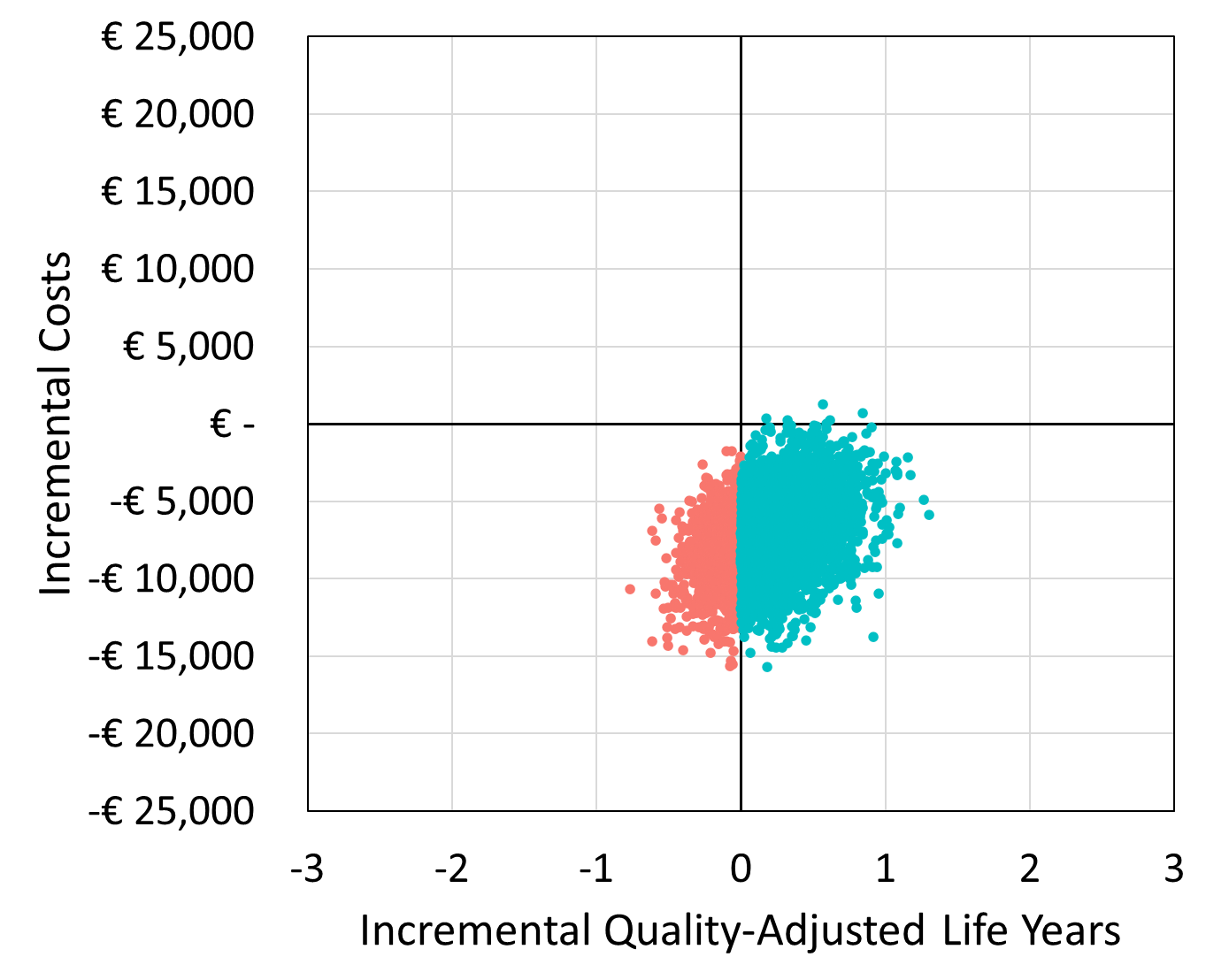
 **A) B)**

Cost-effectiveness planes in the scenario analysis. The graph shows the results of the 5,000 Monte Carlo simulation runs for the probabilistic sensitivity analysis of introducing an active surveillance strategy for women with low-risk DCIS (defined by low COX-2 protein expression and lower relative area of breast adipose tissue) compared to standard surgical intervention. In panel A, effectiveness is represented by incremental quality-adjusted life years, and panel B by incremental life years. Points shown in blue represent simulations in which introducing an active surveillance strategy resulted in overall absolute health benefits across the simulated cohort (Panel A: 80.6%, Panel B: 49.2%)

# Supplementary Figure 4. Cost-effectiveness planes for low-risk subgroups only

*Low-risk defined by ER+ status and low- and intermediate grade*

| **A)**  **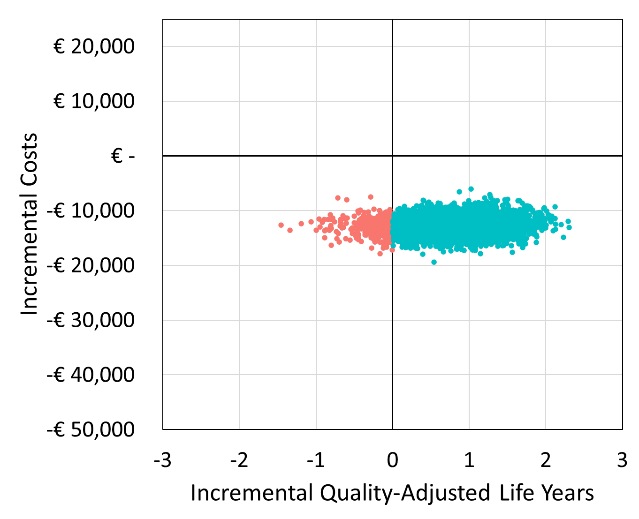** | **B)**  **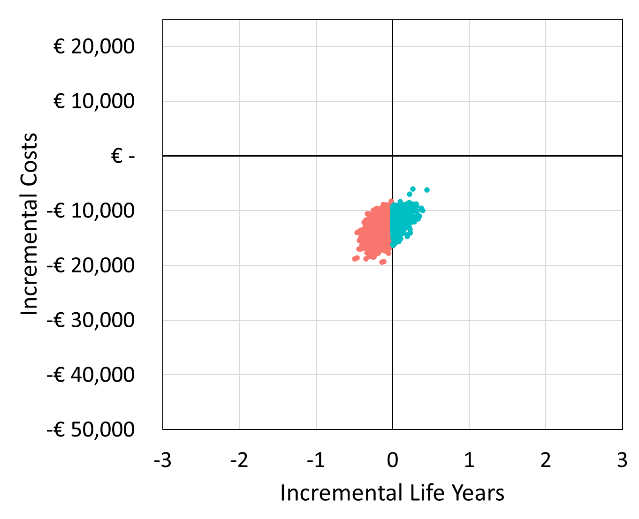** |
| --- | --- |

*Low-risk defined by low COX-2 protein expression and lower relative area of breast adipose tissue*

| **C)**  **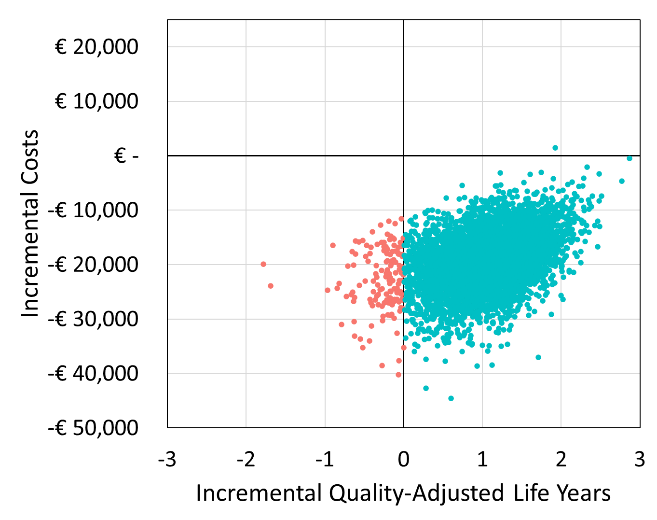** | **D)**  **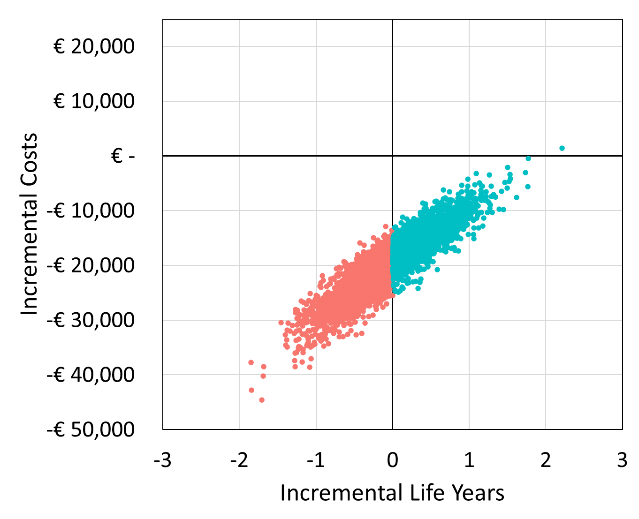** |
| --- | --- |

Cost-effectiveness planes showing the results of shows the results of the 5,000 Monte Carlo simulation runs for the probabilistic sensitivity analysis, using only data on women identified as low-risk. All strategies show cost-savings. Simulations for which an active surveillance strategy resulted in gains in incremental quality-adjusted life years (panels A and C) and incremental life years (panel B and D) are shown in blue (Panel A: 92.9%, Panel B: 18.9%, Panel C: 97.0%, Panel D: 50.5%). The greater the variation of simulation points is an indication of the uncertainty in the parameter inputs used.

# Supplementary Table 6. Probabilistic results, low-risk subgroups only

| **Strategy** | **Incremental costs, 95% CI** | **Incremental life years, 95% CI** | **Incremental QALYs, 95% CI** | **ICER costs per QALY** |
| --- | --- | --- | --- | --- |
| **Low risk defined by ER+ status and low- and intermediate-grade**  **Strategy A:** Standard surgical intervention (BCS±RT)  **Vs.**  **Strategy B:** Active surveillance | -12,715 € (-15,720, -9,597 €) | -0.11 (-0.33, 0.16) | 0.81 (-0.26, 1.70) | -15,788 € |
| **Low-risk defined by low COX-2 protein expression and lower relative area of breast adipose tissue**  **Strategy A:** Standard surgical intervention (BCS±RT)  **Vs.**  **Strategy B:** Active surveillance | -19,128 € (-29,815, -9,490 €) | 0.00 (-0.94, 0.91) | 1.02 (-0.05, 1.98) | -18,698 € |
